# The microRNA miR-30a blocks adipose tissue fibrosis accumulation in obesity

**DOI:** 10.1172/JCI175566

**Published:** 2025-06-05

**Authors:** Pradip K. Saha, Robert Sharp, Aaron R. Cox, Rabie Habib, Michael J. Bolt, Jessica B. Felix, Claudia E. Ramirez Bustamante, Xin Li, Sung Yun Jung, Kang Ho Kim, Kai Sun, Huaizhu Wu, Samuel Klein, Sean M. Hartig

**Affiliations:** 1 Division of Diabetes, Endocrinology, and Metabolism, Department of Medicine and; 2 Department of Molecular and Cellular Biology, Baylor College of Medicine, Houston, Texas, USA.; 3 Center for Metabolic and Degenerative Diseases, the Brown Foundation Institute of Molecular Medicine for the Prevention of Human Diseases, University of Texas Health Science Center at Houston, Houston, Texas, USA.; 4 Advanced Technology Core Labs and; 5 Department of Biochemistry, Baylor College of Medicine, Houston, Texas, USA.; 6 Department of Anesthesiology, Critical Care, and Pain Medicine, McGovern Medical School, University of Texas Health Science Center at Houston, Texas, USA.; 7 Cardiovascular Research, Department of Medicine, Baylor College of Medicine, Houston, Texas, USA.; 8 Center for Human Nutrition, Washington University School of Medicine, St. Louis, Missouri, USA.

**Keywords:** Cell biology, Metabolism, Adipose tissue, Fibrosis, Noncoding RNAs

## Abstract

White adipose tissue (WAT) fibrosis occurring in obesity contributes to the inflammatory and metabolic comorbidities of insulin resistance and type 2 diabetes, yet the mechanisms involved remain poorly understood. Here, we report a role for the broadly conserved miRNA *miR-30a* as a regulator of WAT fibrosis and systemic glucose metabolism. Mice modified to express *miR-30a* at elevated levels in adipose tissues maintain insulin sensitivity coupled with reduced fatty liver disease when fed a high-fat diet. These effects were attributable to cell-autonomous functions of *miR-30a* that potently increase expression of adipocyte-specific genes. Proteomic screening revealed *miR-30a* limits profibrotic programs in subcutaneous WAT, at least in part, by repressing PAI-1, a dominant regulator of fibrinolysis and biomarker of insulin resistance. Conversely, mouse adipocytes lacking *miR-30a* exhibited greater expression of fibrosis markers with disrupted cellular metabolism. Lastly, *miR-30a* expression negatively correlates with *PAI-1* levels in subcutaneous WAT from people with obesity, further supporting an antifibrotic role for *miR-30a*. Together, these findings uncover *miR-30a* as a critical regulator of adipose tissue fibrosis that predicts metabolically healthy obesity in people and mice.

## Abstract 2

Our study used pre-clinical mouse and human models to uncover microRNA regulation of metabolically healthy obesity.

## Introduction

Among the clinical relationships proposed to explain how excess body weight causes insulin resistance, mounting evidence implicates white adipose tissue (WAT) fibrosis, and the abundance of extracellular matrix proteins correlates strongly with obesity and its comorbidities (1). Accumulation of extracellular matrix proteins occurs as part of the microenvironment responses to obesity required for healthy WAT expansion. However, pathologically excessive buildup of extracellular matrix causes fulminant inflammatory cell infiltration and fibrosis. The definitive association between fibrosis in WAT and insulin resistance underscores the importance of understanding the underlying mechanism(s) that contribute to the pathophysiology of obesity.

Weight gain causes WAT expansion, which involves fat cell hypertrophy and subsequent immune cell infiltration. Consistent with this response to obesity stress, increased subcutaneous adipocyte size (hypertrophy) is typically associated with insulin resistance and increased risk of developing type 2 diabetes mellitus (T2DM) independent of total fat mass (2–7). Hypertrophic adipocytes are more likely to increase extracellular matrix protein concentrations and engage fibrosis programs that ultimately limit WAT expansion. Such changes to the extracellular matrix and consequent fibrosis in subcutaneous WAT of people are linked to insulin resistance and T2DM (8). Furthermore, studies in people and mice firmly demonstrated that greater frequencies of less-inflamed, smaller adipocytes, particularly in the subcutaneous WAT, predict metabolically healthy obesity (9). However, the driving forces of adipocyte size and fibrosis responses to obesity remain largely unknown.

miRNAs are noncoding RNAs of 20–25 nucleotides that bind target mRNAs in the 3′ UTR to induce mRNA degradation and inhibit protein translation (10). Preclinical and clinical studies demonstrate miRNAs regulate metabolism in peripheral tissues for the purposes of coordinating energy balance. miRNAs expressed in adipose tissues govern levels of diverse factors that perform and specify fat cell differentiation, as well as secretion of endocrine factors for glucose and insulin sensitivity. More than 40 miRNAs correlate with human obesity and T2DM, and numerous miRNAs affect adipocyte differentiation (11). *miR-30a* is among the most highly expressed miRNAs in WAT, and its expression in subcutaneous fat predicts insulin sensitivity in people and mice (12). In the complex microenvironment of WAT, high *miR-30a* expression suppresses autophagy proteins (13, 14) and inflammatory mediators (12) that correlate with insulin resistance. Conversely, *miR-30a* expression in adipocytes is lower in mice and people with obesity and insulin resistance (12). Here, we generated an adipocyte-specific *miR-30a* transgenic mouse (*miR-30a^fat^*) model to investigate how obesity-resistant *miR-30a* expression in WAT sustains insulin sensitivity. These findings add insight into the regulation of WAT fibrosis in obesity and cellular mechanisms that contribute to systemic metabolic homeostasis.

## Results

### miR-30a transgenic expression promotes adipocyte differentiation.

**Figure 1:**
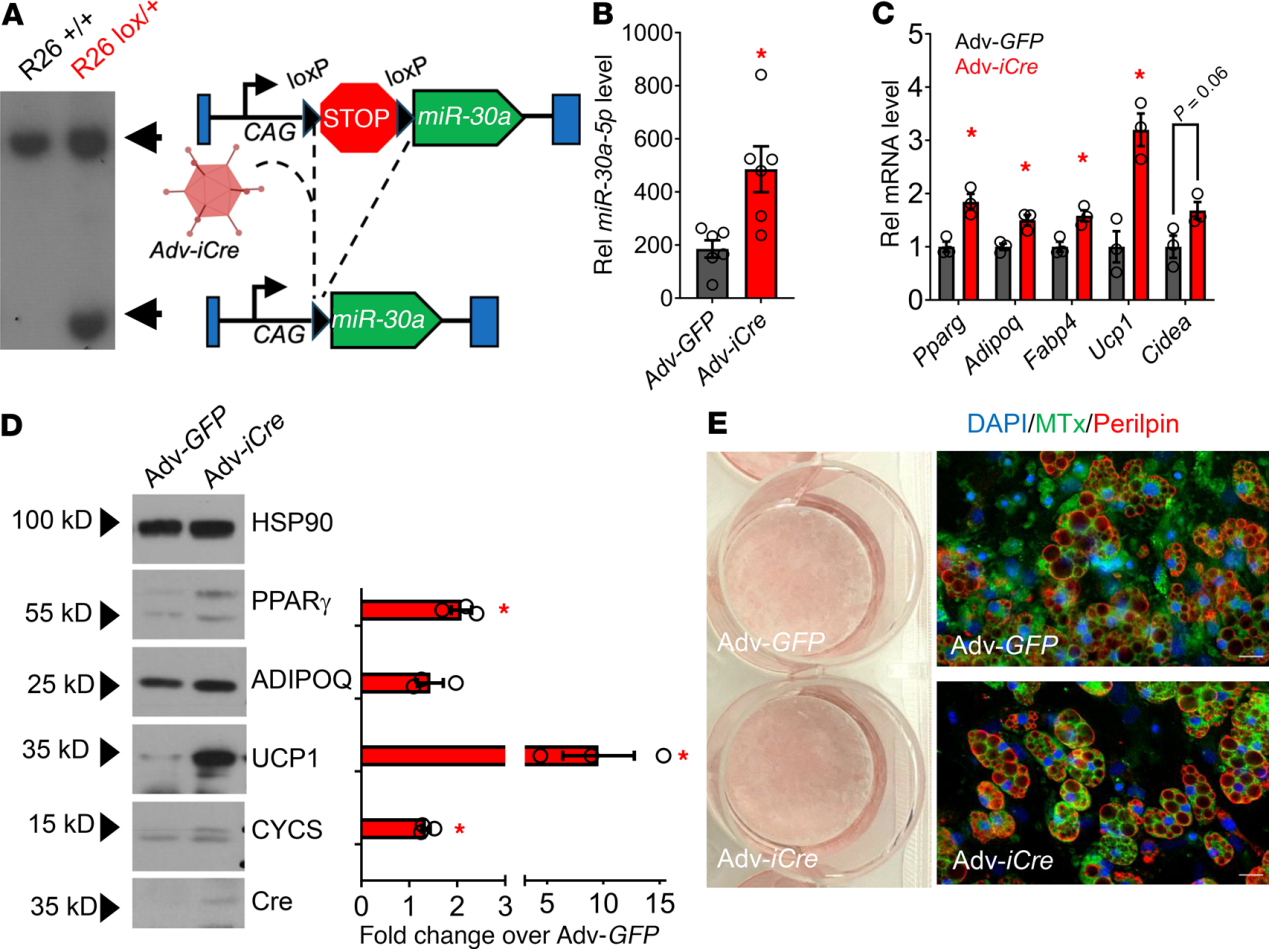
Enforced *miR-30a* expression increases adipocyte differentiation. (**A**) Southern blot was performed with embryonic stem cell genomic DNA digested with EcoRV and 5′ probe. Targeting of the lox-stop-lox–*miR-30a* to the *Rosa26* locus generated 2 bands. (**B**) Adv-iCre deleted the STOP cassette and allowed transgenic *miR-30a* expression in iWAT SVF-derived adipocytes (*n* = 6/group). (**C**) mRNA expression of selected adipocyte differentiation markers (*n* = 3/group). (**D**) Cre immunoblotting was performed and quantified (*n* = 3) to confirm transduction, along with markers of mature fat cells. HSP90 served as the loading control. (**E**) Lipid accumulation within differentiated adipocytes was visualized by ORO for gross effects (left) and deconvolution microscopy (right). Fluorescent dyes are mitochondria (MTx) (green), perilipin (red), and nuclei (DAPI; blue). Scale bars: 20 µm. All data are represented as the mean ± SEM.*P < 0.05, by 2-tailed (**C**) or 1-sided (**D**) unpaired Student’s *t* test versus Adv-GFP. Rel, relative.

We demonstrated that expression of the miRNA, *miR-30a,* in WAT positively correlates with insulin sensitivity in mice and people and that adenovirus (Adv) delivery of *miR-30a* into subcutaneous WAT (i.e., inguinal WAT [iWAT]) enhances insulin sensitivity in mice with diet-induced obesity (12, 15). However, WAT contains many cell types, including adipocytes and immune cells (16, 17). To identify cell types responsible for these metabolic benefits, we created genetically engineered mouse models to test the effects of cell-restricted *miR-30a*. We first created a line harboring the lox-stop-lox *miR-30a* transgene at the ROSA26 locus (*miR-30a^L/L^*) using homologous recombination in C57BL/6J embryonic stem cells, and the presence of loxP sites in the targeted region was confirmed by Southern blot (Figure 1A). We verified that the loxP sites targeted the Rosa26 locus by transducing *miR-30a^L/L^* iWAT-derived, stromal vascular fraction (SVF) fibroblasts with Adv expressing Cre recombinase (Adv-iCre) or GFP (Adv-GFP). Adv-iCre caused almost 3-fold induction of *miR-30a* relative to Adv-GFP controls (Figure 1B).

To explore the effects of transgenic *miR-30a* overexpression in fat cells, we isolated iWAT SVF fibroblasts for in vitro differentiation. After Adv-GFP or Adv-iCre infections and differentiation for 8 days, we observed an increase in several conventional and brown/beige adipocyte marker genes (18), including *Pparg*, *Adipoq*, and *Ucp1* (Figure 1C), consistent with observations from human subcutaneous adipocytes (19). Western blot confirmed Cre expression and induction of mitochondrial proteins (UCP1 and CYCS) in Adv-iCre conditions (Figure 1D). However, Oil Red O (ORO) staining and immunofluorescence to PLIN1 did not distinguish adipocytes expressing *miR-30a* (Adv-iCre) from control cells (Figure 1E). Collectively, these efforts validate the transgene activity and confirm previous roles (19) for *miR-30a* during adipocyte differentiation.

### Adipocyte-specific miR-30a expression uncouples obesity from T2DM in mice.

**Figure 2:**
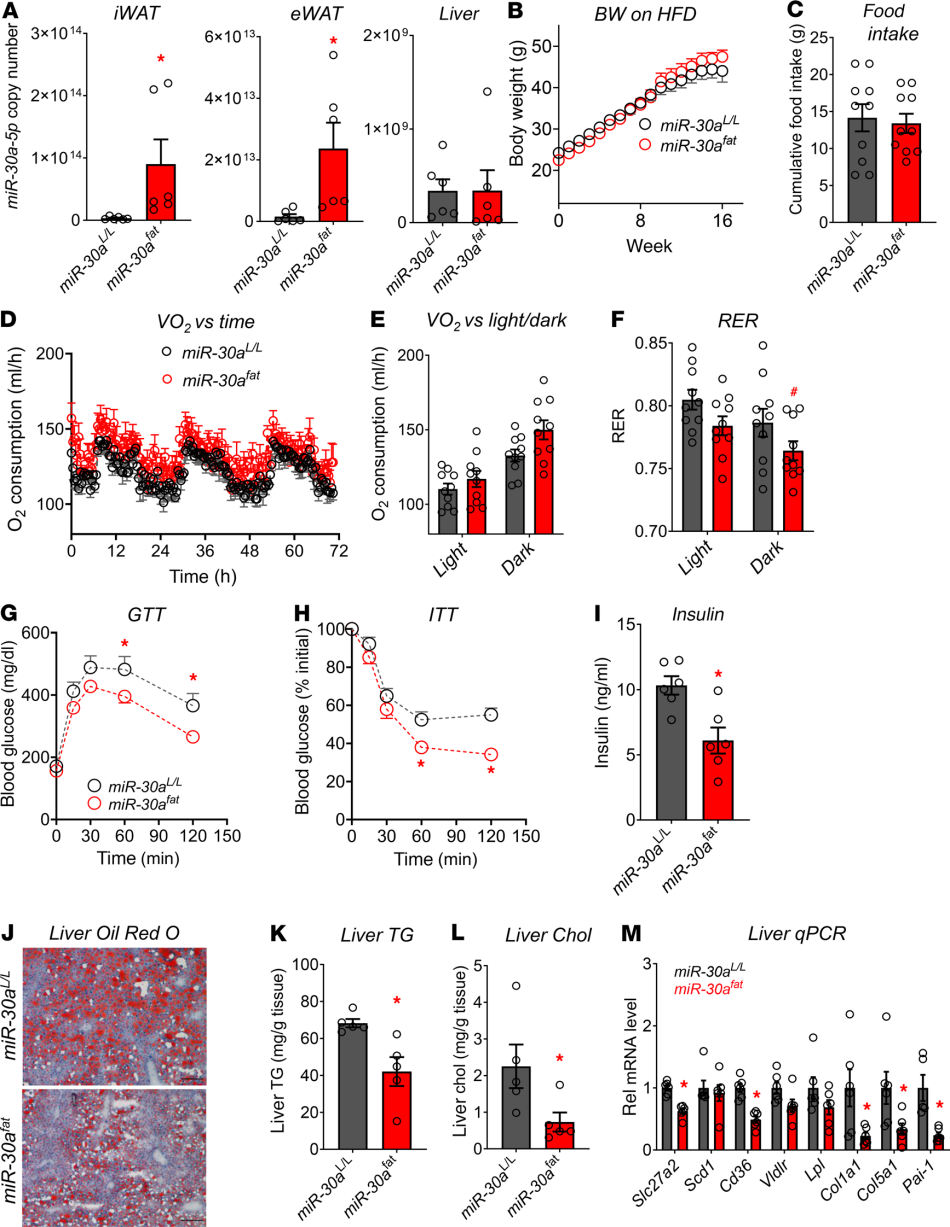
Conditional *miR-30a* transgenesis in WAT maintains insulin sensitivity in obesity. (**A**) Copy number analysis per 10 ng RNA of *miR-30* family members in WAT and liver of *miR-30a^fat^* mice after 18 weeks of an HFD (*n* = 6/group). A log scale is shown. (**B**) Body weight of male *miR-30a^L/L^* and *miR-30a^fat^* mice during HFD feeding (*n* = 10–16/group). Mice were individually housed and monitored in CLAMS-HC metabolic cages for 3 days (*n* = 10/group, unless otherwise noted). (**C**) Cumulative food intake (g) and (**D**) recorded traces of O_2_ consumption (mL/h) (*n* = 5/group). (**E**) Average O_2_ consumption and (**F**) RER during light and dark periods (*n* = 10/group). Data were analyzed with CalR and ANCOVA using lean mass as covariate for O_2_ consumption. (**G**) Glucose (GTT) and (**H**) insulin (ITT) tolerance tests (*n* = 16–21/group) with corresponding (**I**) 4-hour fasting serum insulin (*n* = 6/group) in *miR-30a^L/L^* and *miR-30a^fat^* after 18 weeks of an HFD. (**J**) Liver sections were stained with ORO to analyze steatosis in male *miR-30a^L/L^* and *miR-30a^fat^* mice after HFD feeding. Reduced fat content in the liver (*n* = 5–6/group) was confirmed by measurement of (**K**) hepatic TGs and (**L**) cholesterol (Chol). (M) Quantitative PCR was used to determine the expression of lipogenic genes in the liver (*n* = 5–6/group). All data are represented as the mean ± SEM. *P < 0.05, by 2-tailed, unpaired Student’s *t* test (**A**, **C**, **I**, **K**, **L**, and **M**). *P < 0.05, ^#^*P* < 0.10, by 2-way ANOVA with Tukey’s multiple-comparison test (**B**, **F**, **G**, and **H**). VO_2_, oxygen consumption.

Given that *miR-30a* levels are high in WAT in physiologic conditions (C57BL/6 mice fed standard chow) and reduced with diet-induced obesity (12), we overexpressed *miR-30a* in WAT (*miR-30a^fat^*) by crossing *Adipoq*-Cre mice with *miR-30a^L/L^* mice. Cre-mediated excision of the stop cassette allowed the CAG promoter to drive high levels of *miR-30a* in WAT depots but not in the liver (Figure 2A). In line with our local Adv transgenesis experiments (12), we were surprised that *miR-30a^fat^* and control littermates did not show differences in body weight gain (Figure 2B) during 18-weeks of a high-fat diet (HFD). To explore the metabolic phenotypes of *miR-30a^fat^* mice, we used Comprehensive Lab Animal Monitoring System Home Cages (CLAMS-HC) to measure energy balance. After 18 weeks of an HFD, we did not observe differences in food intake (Figure 2C). Oxygen consumption trended higher in *miR-30a^fat^* mice compared with *miR-30a^L/L^* littermates (Figure 2D), but ANCOVA did not detect differences during light and dark phases (Figure 2E) between groups when accounting for lean body mass. To examine whether obese *miR-30a^fat^* and littermate control mice displayed different metabolic substrate preferences, we extracted the respiratory exchange ratio (RER) during dark and light periods. When compared with *miR-30a^L/L^* controls, *miR-30a^fat^* mice had lower RER values in the dark phase (Figure 2F) pointing to preferential use of fat as a metabolic substrate in the transgenic animals.

Although weight gain and energy expenditure were not altered among obese *miR-30a^fat^* mice and *miR-30a^L/L^* littermates, *miR-30a* expression in fat cells improved insulin sensitivity. *miR-30a^fat^* considerably augmented glucose (Figure 2G) and insulin (Figure 2H) tolerance. Fasted serum insulin levels (Figure 2I) were also significantly decreased. As a result, greater insulin sensitivity in obese *miR-30a^fat^* mice led to concurrent reductions in gross measures of fatty liver disease associated with the HFD (Figure 2J), including decreased hepatic triglyceride (TG) (Figure 2K) and cholesterol (Figure 2L) levels, as well as expression of genes (Figure 2M) associated with fatty acid transport (*Slc27a2*, *Cd36*) and fibrosis (*Pai-1*, *Col1a1*, *Col5a1*). Collectively, these data indicate that continuous *miR-30a* expression in adipocytes preserves systemic insulin sensitivity after an HFD without altering body weight.

### Sustained miR-30a expression in WAT reduces subcutaneous adipocyte cell size in obese mice.

**Figure 3:**
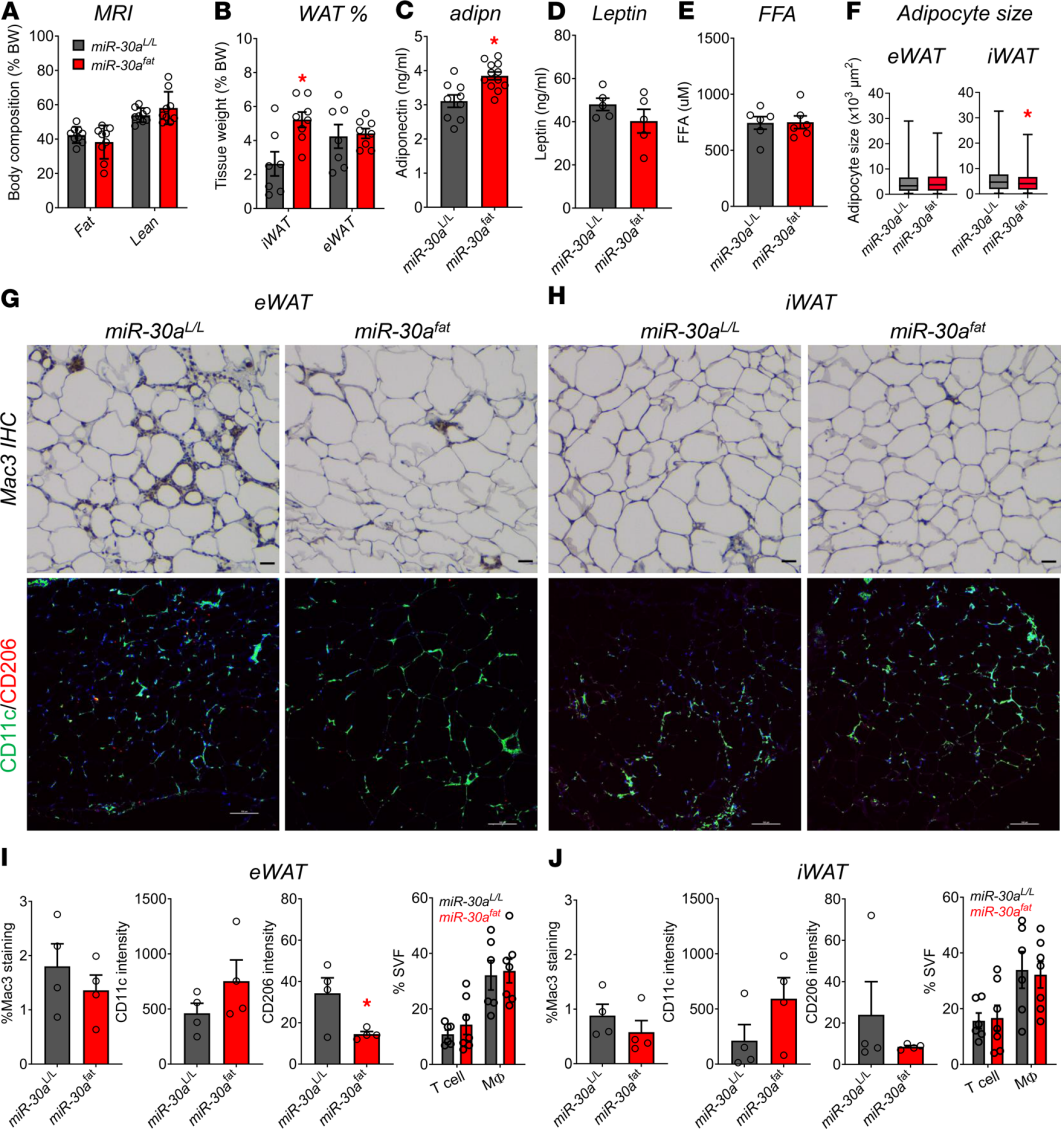
*miR-30a* expression expands iWAT during HFD feeding. (**A**)Mice fed an HFD for 18 weeks underwent MRI (*n* = 10/group) to measure whole-body lean and fat mass. (**B**) Tissue weights from male *miR-30a^L/L^* and *miR-30a^fat^* mice (*n* = 7–8/group) at necropsy. Serum levels of adiponectin (adipn) (*n* = 9–12) (**C**), leptin (*n* = 5) (**D**), and free fatty acid (FFA) (*n* = 6) (**E**) after feeding. (**F**) Mean adipocyte size (μm^2^) measured across 4 fields of view (*n* = 3/group) from (**G**) eWAT and (**H**) iWAT sectioning and immunohistochemistry. (**G** and **H**) WAT was stained for Mac3 (upper rows; scale bars: 50 μm) or CD11c and CD206 (lower rows; scale bars: 100 μm). (**I**) eWAT and (**J**) iWAT quantification of Mac3 staining (%area; *n* = 4/group) or CD11c and CD206 intensities (*n* = 4/group). (**I** and **J**) The bar charts also include analysis of T cells and macrophages in the WAT SVF quantified by flow cytometry (*n* = 6–7 mice/group). All data are represented as the mean ± SEM. *P < 0.05, by 2-way ANOVA with Tukey’s multiple-comparison test (**A** and **B**). *P < 0.05, by 2-tailed, unpaired Student’s *t* test (**C**–**F**, **I**, and **J**).

To further understand how the WAT of *miR-30a^fat^* mice responded to the HFD, we analyzed body composition and adipocyte-specific serum markers. Although no differences in total fat and lean mass were detected after being fed an HFD (Figure 3A), sustained *miR-30a* in adipocytes caused a selective increase in iWAT mass (Figure 3B). Additionally, we found adipose-specific transgenic *miR-30a* expression caused increases in serum adiponectin (Figure 3C) independent of meaningful changes in circulating leptin (Figure 3D) and free fatty acid (Figure 3E) levels in HFD-fed *miR-30a^fat^* mice compared with littermate controls.

Diet-induced obesity increases macrophage and T cell infiltration of epididymal WAT (eWAT) and iWAT depots, which correlates with impaired WAT expansion and bigger fat cell size (17). Previous studies (12) established adipocyte-specific expression of *miR-30a* decreased the production and impacts of pro-inflammatory signaling molecules in WAT and thereby restored metabolic fitness in fat cells. To extend these data, we performed histology and flow cytometry studies of iWAT and eWAT excised from *miR-30a^fat^* mice and littermate controls. Quantitative image-based histological analysis revealed that adipocytes from *miR-30a^fat^* iWAT were significantly smaller (Figure 3F), favoring reduced adipocyte hypertrophy (20). However, blinded immunohistochemistry studies showed that obese *miR-30a^fat^* and *miR-30a^L/L^* mice accumulated variable patterns of Mac3 and other macrophage markers in the eWAT (Figure 3G) and iWAT (Figure 3H). Likewise, CD11c (green) staining in WAT depots was indistinguishable between *miR-30a^fat^* and *miR-30a^L/L^* littermates. CD206 (red) staining was also detected, albeit at very low levels, and trended lower in obese *miR-30a^fat^* mice in the eWAT. These observations were confirmed by quantitative analysis of Mac3, CD11c, and CD206 staining in eWAT (Figure 3I) and iWAT (Figure 3J). We also confirmed T cell and macrophage numbers in WAT, using standard antibody markers and flow cytometry analysis of immune cells extracted from the SVF. eWAT (Figure 3I) and iWAT (Figure 3J) T cell and macrophage populations were not altered in either mouse genotype, suggesting canonical adipose tissue inflammatory cells did not have a meaningful influence on whole body metabolism in *miR-30a^fat^* mice. These results establish *miR-30a* transgenic expression in *Adipoq*^+^ cells expands iWAT mass and adipocyte functions, largely independent of canonical pro-inflammatory responses to obesity.

### Diet-induced subcutaneous WAT fibrosis is repressed by miR-30a.

**Figure 4:**
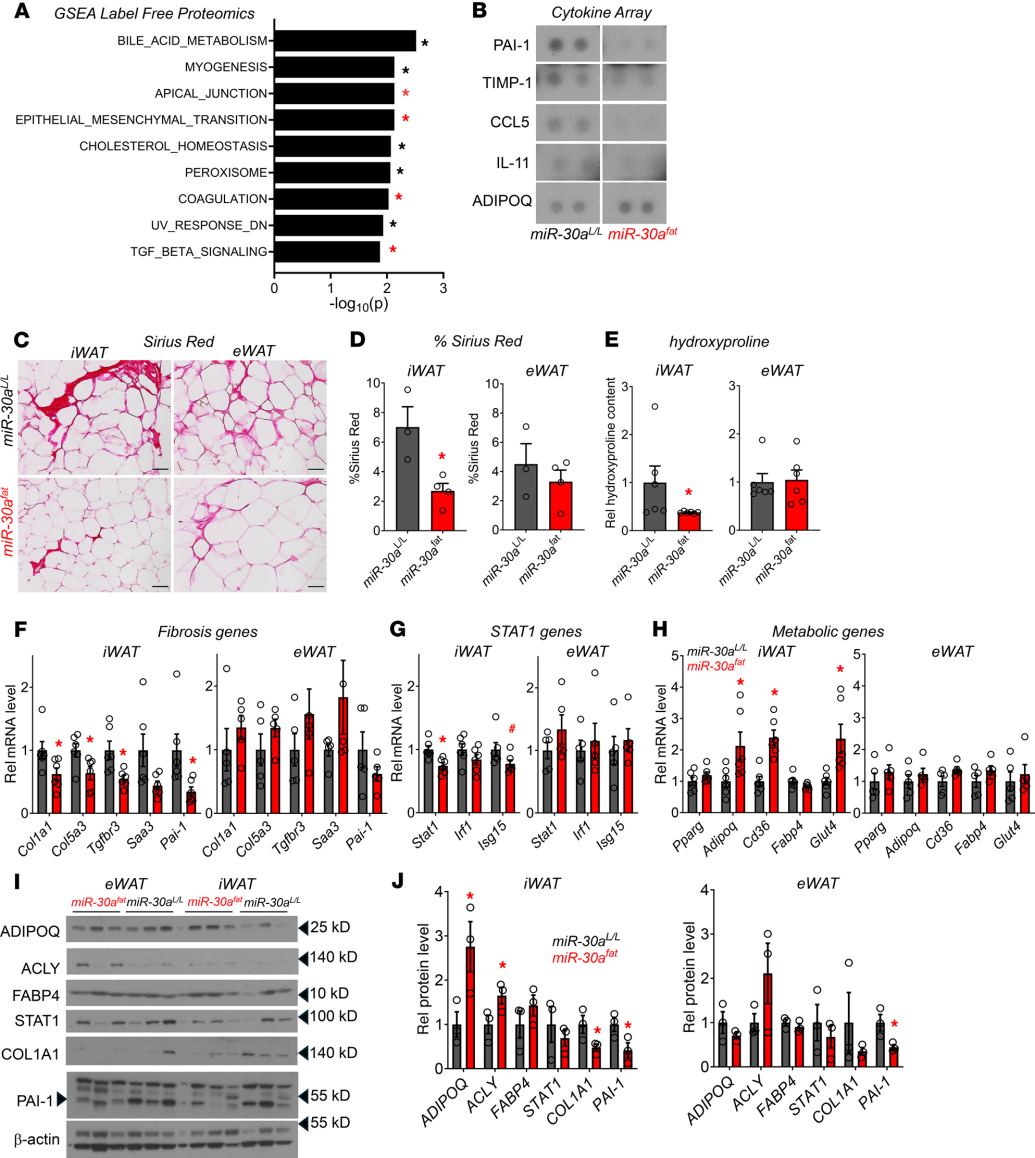
Local antifibrotic effects associated with enforced *miR-30a* expression in subcutaneous WAT of obese mice. (**A**) Gene set enrichment analysis (GSEA) of altered proteins (*P < 0.05 for *miR-30a^fat^*/*miR-30a^L/L^*; *n* = 4/group) identified signatures depleted by transgenic *miR-30a* expression in the iWAT of obese mice. (**B**) iWAT protein lysates (pooled *n* = 4/group) were incubated with cytokine arrays to follow up the proteomic screen. (**C**) Sirius red staining in the iWAT and eWAT after HFD feeding for 18 weeks. Scale bars: 50 μm. eWAT and iWAT (**D**) quantification of percentage of Picrosirius red staining (%area; *n* = 3–4/group) and hydroxyproline content (**E**) by mass spectrometry (*n* = 6/group, relative to *miR-30a^L/L^*). Expression profiles of profibrotic (**F**), STAT1 targets (**G**), and metabolic genes (**H**) (*n* = 6/group) in the iWAT and eWAT after HFD feeding for 18 weeks (n = 6/group). (**I**) Western blotting with indicated antibodies and associated quantification (**J**) to validate changes in fibrosis markers with independent WAT protein lysates. HSP90 and β-actin served as invariant protein controls. All data are represented as the mean ± SEM. *P < 0.05 and ^#^*P* < 0.10, by 2-tailed, unpaired Student’s *t* test (**D**–**H** and **J**). Rel, relative.

The metabolic effects we observed in *miR-30a^fat^* mice corresponded with smaller adipocyte size, which correlates with insulin sensitivity (9). However, the mechanisms that explain how *miR-30a* exerts metabolic benefits were not clear. To identify targets of transgenic *miR-30a* expression in WAT, we used proteomic profiling to nominate any pathways that defined the effects of *miR-30a* expression in adipocytes. We detected 299 unique proteins reduced in *miR-30a^fat^* iWAT compared with *miR-30a^L/L^*. Among proteins lowered in the iWAT of transgenic mice, the most enriched miRNA targets were those of *miR-30a* (44 *miR-30a* targets of 299 total proteins reduced; *P* < 0.0001). Furthermore, *miR-30a* broadly suppressed proteins enriched for epithelial mesenchymal transition, TGF-β signaling, and coagulation pathways (Figure 4A) frequently associated with myofibroblast recruitment (8). Follow-up studies used adipokine arrays for targeted validation of epithelial mesenchymal transition and other fibroblast responses. Blocking these pathways coincided with decreased levels of pro-fibrotic molecules (PAI-1, TIMP-1) and some chemokines (IL-11 and CCL5) in iWAT (Figure 4B). These data were consistent with *miR-30a* targets (ELMOD2, DOCK7, GFPAT2, LGPAT1 SMAD1, UNC119) and other extracellular matrix proteins (COL5A3, COL5A1, COL1A1, COL14A1, COL6A6, FBLN2, ITGB4, SAA2) depleted in the proteomic screen of iWAT known to promote fibrosis in metabolic organs (21–28).

In people with obesity, fibrosis limits subcutaneous adipose tissue expansion capacity and predicts insulin resistance and fatty liver disease (29–33). Based on our proteomic studies, adipocyte-specific expression of *miR-30a* may decrease fibrosis in iWAT, which, in turn, correlates with protection from insulin resistance and fatty liver disease. Grossly, we found fewer fibrotic structures (Figure 4C) and less Sirius red staining (Figure 4D) in obese *miR-30a^fat^* mice. Likewise, we found reduced hydroxyproline content (Figure 4E) and mRNA expression of several fibrosis marker genes (*Col1a1*, *Col5a3*, *Tgfbr3*, *Saa3*, *Pai-1*) in iWAT of *miR-30a^fat^* mice compared with controls (Figure 4F). Targeted quantitative PCR (qPCR) analysis of inflammatory (Figure 4G) and adipocyte-specific genes (Figure 4H) also validated previous studies (12), extending the concept that *miR-30a* expression promotes metabolic competence in fat cells. Lastly, Western blots (Figure 4I) confirmed the depletion of fibrosis proteins (PAI-1, COL1A1) associated with higher levels of adipocyte-specific proteins (ADIPOQ, ACLY) exclusively in the iWAT of *miR-30a^fat^* mice (Figure 4J).

### PAI-1 can be regulated by miR-30a in adipocytes.

**Figure 5:**
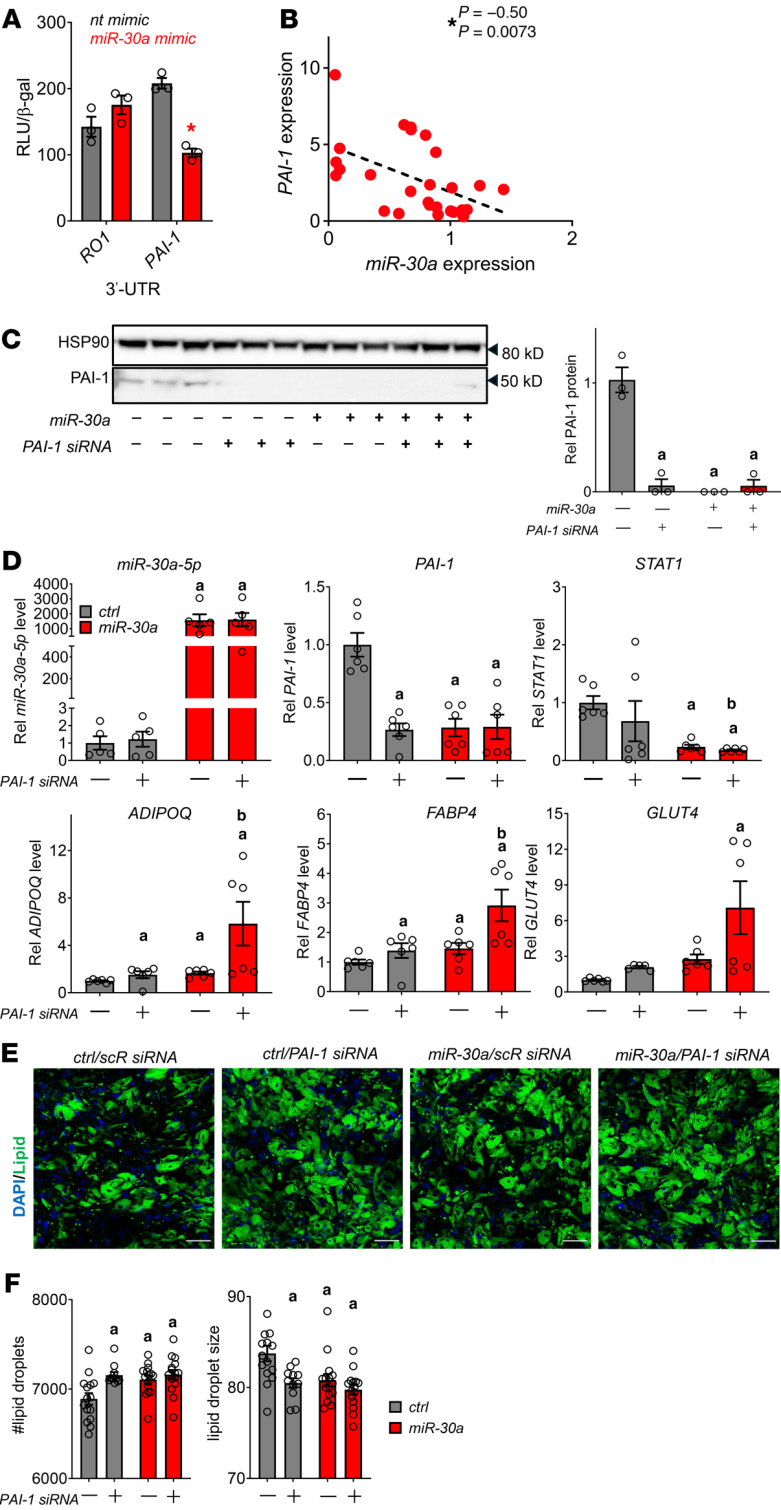
*PAI-1* is a direct target of *miR-30a*. (**A**) Plasmids with negative control (RO1) and *PAI-1* 3′ UTR luciferase fusions were cotransfected with *miR-30a* or control (nt) mimics in human adipocytes. *P < 0.05, by 2-way ANOVA with Tukey’s multiple-comparison test. (**B**) Relative *miR-30a* and *PAI-1* expression measured in subcutaneous adipose tissue from humans. *P value for Pearson’s *r* < 0.05 by *t* test performed on the linear regression. (**C**–**F**) Human adipocytes were transfected with siRNA to *PAI-1* ± *miR-30a* mimics for 48 hours. (**C**) Western blotting and with PAI-1 antibodies and associated quantification. HSP90 served as invariant protein controls. (**D**) mRNA expression of selected adipocyte marker genes, *STAT1*, and *PAI-1* in adipocytes (*n* = 5–6/group). (**E**) Representative images from high-throughput microscopy (HTM) following immunofluorescence labeling of lipid droplets (green, BODIPY) and nuclei (blue, DAPI) of human adipocytes treated with siRNA to PAI-1 ± *miR-30a* mimics. Scale bars: 50 µm. (**F**) High-content analysis of differentiated adipocytes from HTM: number of lipid droplets and mean lipid droplet size (*n* = 12–15 replicates/group). All data are represented as the mean ± SEM. *P* < 0.05 versus ^a^control mimics/scRNA and ^b^transfection of individual siRNA *PAI-1* or *miR-30a* mimics, by 2-way ANOVA with Tukey’s multiple-comparison test (**C**, **D**, and **F**). Ctrl, control; nt,nontargeting.

We explored direct connections between *miR-30a* and mediators of fibrosis using databases of miRNA-target binding inferred by high-throughput sequencing (10, 34–36) and found consistent targeting of the *PAI-1* 3′-UTR by *miR-30a*. To validate *miR-30a* putative interactions with *PAI-1* 3′-UTR, we cotransfected miRNA mimics and UTR-luciferase reporter fusions in human adipocytes. As predicted, *miR-30a* expression decreased relative luciferase activity of the *PAI-1* 3′-UTR. In contrast, luciferase activity of control 3′-UTR fusions (RO1), which lack any pertinent miRNA binding sites, remained unchanged by *miR-30a* or control mimic transfections (Figure 5A). Consistent with these findings, *miR-30a* levels were inversely correlated with *PAI-1* mRNA in subcutaneous WAT biopsy specimens from people with obesity (Figure 5B).

To examine the extent to which *miR-30a* targeting of PAI-1 caused changes to fat cells associated with differentiation and overall energy accretion, we transfected human adipocytes with *miR-30a* mimics, siRNA to *PAI-1*, combined *miR-30a* mimics and siRNA to *PAI-1*, or appropriate controls (Figure 5C). As expected, siRNA to *PAI-1*, *miR-30a* mimics, or combined transfections depleted PAI-1 protein expression by more than 90%. We also found *PAI-1* knockdown exhibited higher metabolic gene expression in similar ways as *miR-30a* expression. Importantly, we validated *miR-30a* depleted *STAT1* and *PAI-1* levels. *miR-30a* expression on top of *PAI-1* siRNA increased levels of metabolic genes above individual transfections (Figure 5D).

To further identify cellular changes associated with *PAI-1* knockdown in adipocytes, we used high-throughput spinning disk confocal microscopy and CellProfiler (37) to extract quantitative features informing lipid accumulation (Figure 5E). Consistent with the smaller fat cells in *miR-30a^fat^* mice (Figure 3F), human adipocytes transfected with *PAI-1* siRNA or *miR-30a* mimics accumulated more lipid droplets of reduced size (Figure 5F). We also performed cotransfection studies and found combined *PAI-1* depletion and *miR-30a* overexpression conditions did not additively influence the number or size of lipid droplets, suggesting shared pathway effects (Figure 5F). Overall, these data demonstrate sustained expression of *miR-30a* exhibits similar functions as *PAI-1* depletion in that it supports metabolic changes in adipocytes strongly associated with insulin sensitivity.

**Figure 6:**
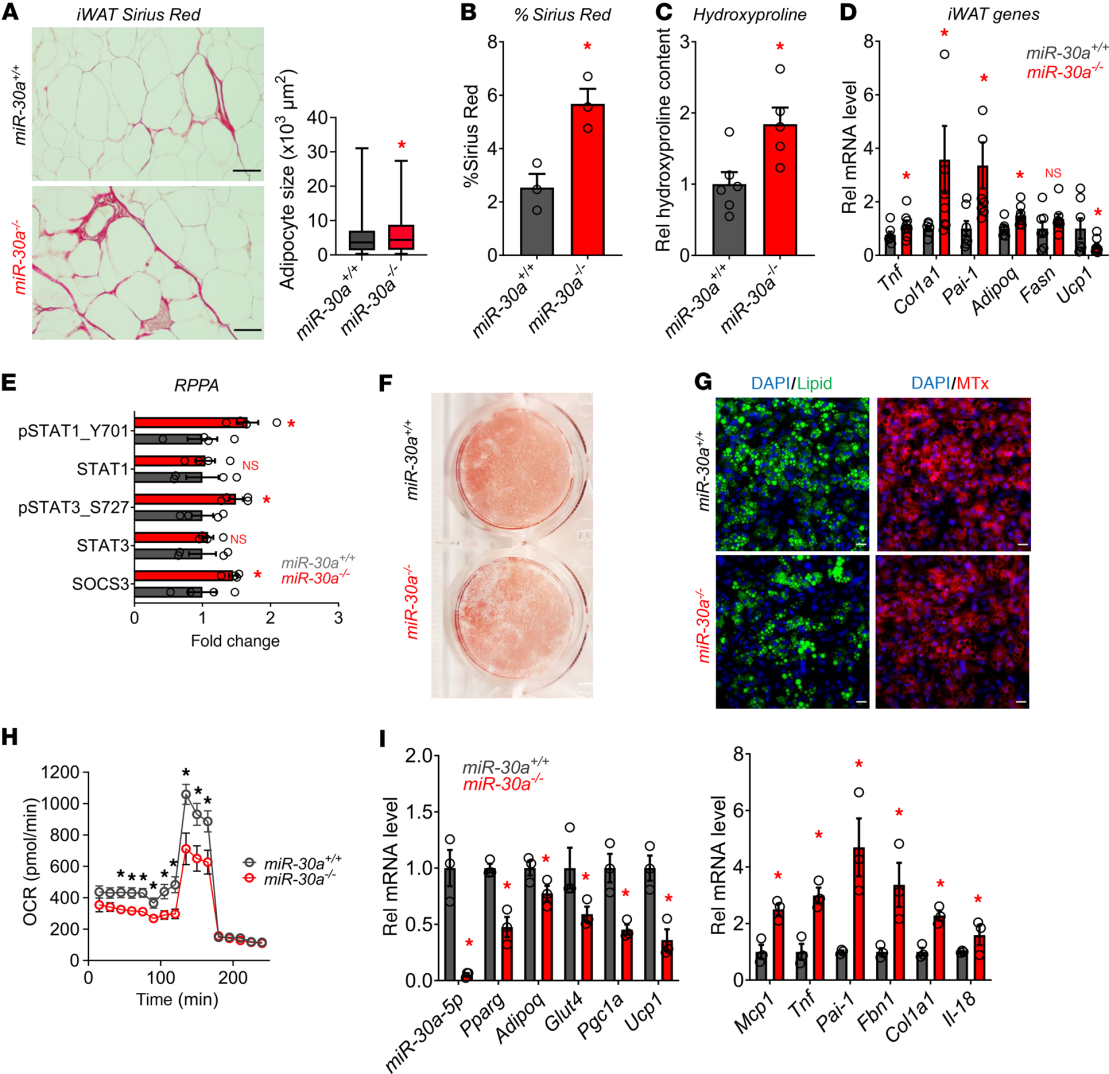
Knockout of *miR-30a* de-represses fibrosis genes and blocks adipocyte differentiation. (**A**) iWAT Sirius red staining and mean adipocyte size (μm^2^) measured across 4 fields of view (*n* = 3/group) of *miR-30a^–/–^* or *miR-30a^+/+^* littermate controls after HFD feeding for 12 weeks. Scale bars: 50 μm. (**B**) Quantification of percentage of Picrosirius red staining (%area; *n* = 3/group, relative to *miR-30a^+/+^*). (**C**) Hydroxyproline content by mass spectrometry (*n* = 5–6/group). (**D**) Expression profiles of pro-fibrotic and metabolic genes (*n* = 7–8/group) in the iWAT of *miR-30a^–/–^* or *miR-30a^+/+^* littermate controls after HFD feeding for 12 weeks. (**E**) Reverse phase protein array (RPPA) analysis performed on iWAT shown as fold change *miR-30a^–/–^*/*miR-30a^+/+^*. SVF-derived adipocytes were prepared from *miR-30a^+/+^* and *miR-30a^–/–^* mice. (**F**) Differentiated *miR-30a^+/+^* and *miR-30a^–/–^* cells were stained with ORO to characterize lipid accumulation. (**G**) Adipocytes were stained and imaged using deconvolution microscopy to identify mitochondria (red), lipid (green), and nuclei (blue). Scale bars: 50 µm. (**H**) Oxygen consumption rate (OCR) in differentiated *miR-30a^+/+^* and *miR-30a^–/–^* mice with addition of oligomycin, carbonyl cyanide-4-(trifluoromethoxy) phenylhydrazone and antimycin-A/rotenone (*n* = 4/group). All data are represented as the mean ± SEM. *P < 0.05, by 2-way ANOVA with Tukey’s multiple-comparison test. (**I**) mRNA expression of selected adipocyte differentiation and fibrosis markers in SVF-derived adipocytes (*n* = 3/group). *P < 0.05, by 2-tailed, unpaired Student’s *t* test (**A**–**E** and **I**).

Additionally, our data pointed to the reciprocal concept that *miR-30a* knockout (*miR-30a^–/–^*) increases fibrosis in iWAT after HFD feeding in mice. Although weight gain and energy balance were largely unaltered after 12 weeks of HFD between *miR-30a^–/–^* and wild-type littermates (38), we observed more Sirius red staining of iWAT accompanied by slightly larger adipocytes in *miR-30a^–/–^* mice (Figure 6A). Quantitative measurements of Sirius red staining (Figure 6B) and hydroxyproline content (Figure 6C) confirmed *miR-30a* knockout was associated with increased fibrosis in iWAT. Moreover, we validated the fibrosis genes *Pai-1* and *Col1a1*, and both showed significantly higher levels in the iWAT of *miR-30a^–/–^* mice (Figure 6D). Similar to gene expression profiles of *miR-30a^–/–^* mice, reverse phase protein arrays revealed *miR-30a* ablation increased levels of pro-inflammatory STAT1 (pSTAT1 Y701) and STAT3 (pSTAT3 S727) signaling in iWAT compared with wild-type mice fed the HFD (Figure 6E).

The absence or inhibition of PAI-1 promotes adipocyte differentiation (39, 40). Therefore, we hypothesized that *miR-30a* knockout in adipocytes allows sustained fibrosis gene expression and altered differentiation. To test this prediction and recapitulate the observations in vivo, we examined the consequences of *miR-30a* deletion on adipocyte differentiation using iWAT SVF cells from age- and sex-matched littermates. *miR-30a^–/–^* resulted in blunted differentiation (Figure 6F) and fewer lipid droplets (Figure 6G). MitoTracker staining appeared less abundant and intense compared with *miR-30a^+/+^* control adipocytes (Figure 6G). Additionally, the reduced differentiation phenotype of *miR-30a^–/–^* adipocytes featured diminished oxygen consumption rates (Figure 6H). Lower expression of mature fat cell marker genes was also observed in *miR-30a^–/–^* adipocytes relative to *miR-30a^+/+^* controls (Figure 6I). In line with the regulation of *Pai-1* abundance by *miR-30a* and *Pai-1*, *Pai-1* expression was elevated in *miR-30a^–/–^* adipocytes (Figure 6I). Furthermore, *miR-30a* depletion increased expression of other fibrosis markers, including *Col1a1* and *Fbn1*, as well as the pro-inflammatory cytokines *Mcp1* and *Tnfa* (Figure 6I). These data further support a cell-autonomous role for *miR-30a* in suppressing PAI-1 and the fibrosis that occurs in the subcutaneous WAT of obese mice and people.

## Discussion

In this study, we provide new lines of evidence to describe how *miR-30a* expression in adipocytes sustains insulin sensitivity in mice with diet-induced obesity. Countless experiments in preclinical animal models and descriptive studies in people implicated higher levels of cytokines and mediators of inflammation in WAT cause obesity and insulin resistance. More recently, refined studies in people with obesity demonstrated subcutaneous adipose tissue fibrosis predicts insulin resistance and fatty liver disease (29–33). Our new observations define molecular events that help explain how increased extracellular matrix production in subcutaneous WAT can be tempered to maintain insulin sensitivity in obesity. At the molecular level, *miR-30a* likely targets PAI-1 to limit the pro-fibrotic programs that would otherwise restrict subcutaneous WAT expansion and decrease insulin sensitivity. These studies, therefore, establish a previously uncharacterized *miR-30a*/*Pai-1* interaction as a major regulator of WAT expansion and whole-body metabolism.

Elevated levels of cytokines like PAI-1 characterize the obese adipose tissue microenvironment and do not allow adipocytes to continuously respond to the stress of an HFD (39, 40). Although it is known that one of the primary sources of PAI-1 is the SVF within the WAT, we do not know which specific cell lineage gives rise to PAI-1 and whether there are differential metabolic effects of PAI-1 based on its origin. Our data suggest *Pai-1* inhibition by *miR-30a* occurs in fat cells, because *miR-30a* knockout promotes *Pai-1* de-repression, which corresponds with the suppression of genes essential for lipid and glucose homeostasis in adipocytes. It will now be important to determine whether *miR-30a* or compensation from *miR-30c*, also located on mouse chromosome 1 and retained in *miR-30a^–/–^* (38), blunt combined effects of other obesity-related pro-fibrogenic and inflammatory cytokines that contribute to pathological subcutaneous WAT expansion.

The fatty liver and insulin sensitivity phenotypes of *miR-30a^fat^* mice resemble metabolically healthy obesity in people, a condition characterized by normal clinical measures of metabolic function (e.g., insulin sensitivity and fatty liver) and associated with expansion of subcutaneous WAT depots (41). In contrast, subcutaneous WAT from people with metabolically unhealthy obesity is characterized by hypertrophic (larger) adipocytes, tissue hypoxia and fibrosis, and pro-inflammatory macrophage infiltration (42). In addition, the metabolically unhealthy obesity phenotype is associated with higher serum PAI-1 concentration and fatty liver disease (29–32). Despite a clinical awareness of people with metabolically healthy obesity phenotypes, who account for 20%–30% of the obese population (43), we lack an understanding of factors and mechanisms that explain how excess subcutaneous WAT exerts protective effects in these individuals. Here, we generated a mouse model that adds to only a handful of existing experimental systems (44, 45) to understand the molecular pathways that uncouple obesity from insulin resistance in metabolically healthy obese people. We demonstrated that *miR-30a* increased the abundance of smaller adipocytes in subcutaneous WAT after HFD feeding, which predicts insulin sensitivity in obesity (9). Although effect sizes in eWAT of the *miR-30a^fat^* mice trended lower, we cannot rule out the possibility *miR-30a* expression may affect visceral adipose tissue fibrosis. Subcutaneous and visceral adipose depots have numerous distinctions, including divergent implications for metabolic risk as well as the ability to express UCP1 and the beige fat thermogenic program (46). Future studies examining whether *miR-30a* has the capacity to selectively drive diet-induced hyperplastic effects in WAT depots using other tissue-restricted Cre transgenes should identify mechanisms that contribute to metabolically healthy obesity. Nonetheless, the strong association of subcutaneous fat cell size and intrinsic hyperplastic potential with insulin resistance in people (2–7) point to mechanisms gated by *miR-30a* in peripheral adipose tissue to generate context-specific responses to obesity.

Obesity causes a complicated mixture of immune and stromal interactions with fat cells that define protective responses of WAT to overnutrition. Though inflammation critically enables adipose tissue responses to whole-body energy demands, causal effects on insulin resistance remain debated. Many rodent studies demonstrate that in obesity, WAT immune cells exhibit changes in numbers and phenotypes and contribute to local and systemic insulin resistance (47–49). Other studies using mouse models found insulin resistance causes infiltration of macrophages in WAT (50) and inflammatory responses to obesity remodel adipose tissues in metabolically protective ways (51, 52). In people, WAT immune cells and phenotypes correlate with body mass index and insulin resistance (48, 49, 53–55) but lack clear and causal influences on insulin resistance and T2DM (56). We did not find that *miR-30a^fat^* altered WAT macrophages and T cell abundance after an HFD. However, chronic inflammation also precedes fibrosis, which limits adipose tissue expansion capacity and strongly predicts insulin resistance and fatty liver in people with obesity (30). Our findings implicate that persistent *miR-30a* expression in adipocytes may dampen local PAI-1 and fibrogenesis and allow WAT expansion, even under the stress of obesity.

Limitations of our study include the fact that *miR-30a* may disrupt expression of other predicted targets to synergize with PAI-1 depletion for antifibrotic effects in WAT. miRNAs evolved to exert their biological function by modulating the coordinated expression of large gene networks (57). Consistent with this function, a liberal survey nominated more than 2,400 targets of *miR-30a* (15), and in the present study, we identified 44 candidate proteins attenuated in the iWAT of *miR-30a^fat^* mice. A handful of the targets have reported roles in fibrosis (21–27). In addition, other collagen and extracellular matrix proteins were strongly repressed by *miR-30a* in iWAT (COL5A3, COL5A1, COL1A1, COL14A1, COL6A6, FBLN2, ITGB4, SAA2). Although it is certainly possible that suppression of other targets could contribute to the antifibrotic and insulin sensitivity effects of *miR-30a* expression, no other singular, informative pathway enriched among suppressed proteins in the proteomic screen. Further studies using *miR-30a* conditional knockouts may shed light on other tissue-specific responses and targets. Finally, our data were primarily collected from mice, but the known relationships of *miR-30a* expression in adipocytes and whole-body insulin sensitivity in people (12, 15, 58–60) justify future studies to examine if any targets of *miR-30a* influence WAT fibrosis and cause insulin resistance in obesity.

## Methods

### Sex as a biological variable.

Sexual dimorphism was not a major focus of this study. Our work only investigated metabolic phenotypes in male mice because female mice accumulate less WAT inflammation and fibrosis compared with male mice after HFD feeding (61). It is not known whether the mechanisms observed here occur similarly in female mice. Gene expression studies using RNA from people with obesity included men and women but were not discretized by sex, because we used prospective samples made available for this study. However, higher subcutaneous adipose fibrosis and PAI-1 levels correlate with insulin resistance in men and women with obesity (29–32). Future studies are needed to determine whether *miR-30a* expression in adipocytes causes comparable outcomes in female mice and predicts insulin resistance in women with obesity.

### Animal models and housing.

To develop a mouse model in which *miR-30a* is expressed at elevated levels in adipose tissues (*miR-30a^fat^*), we created a mouse with a lox-stop-lox miR-30a minigene (62) at the ROSA26 locus (*miR-30a^L/L^*) using homologous recombination in C57BL/6J embryonic stem cells. We cloned the primary *miR-30a* coding DNA fragment into a modified version of pROSA26-1 (Addgene, 11739). Gene targeting and production of chimeras were conducted according to established protocols in the Baylor College of Medicine Genetically Engineered Rodent Models Core. Chimeras were bred with C57BL/6 mice screened by PCR genotyping and confirmed by Southern blotting on genomic DNA. Founder lines were backcrossed to C57BL/6 mice for 6 generations. *miR-30a^L/L^* mice were crossed with *Adipoq*-Cre (The Jackson Laboratory, 028020) to generate *miR-30a^fat^* and littermate controls (*miR-30a^L/L^*). *miR-30a^+/+^* and *miR-30a^–/–^* mice were used as previously described (38).

Animal procedures were approved by the Institutional Animal Care and Use Committee of Baylor College of Medicine. All experiments were conducted using littermate-controlled male mice aged 6–8 weeks housed in a barrier-specific, pathogen-free animal facility with a 12-hour dark-light cycle and free access to water and food. At the end of the experiments, mice were euthanized by cervical dislocation while under isoflurane anesthesia. After euthanasia, tissues were collected, flash-frozen in liquid N_2_, and stored at –80°C until use. All experiments adhered to Animal Research: Reporting of in vivo Experiments (ARRIVE) guidelines.

### Human adipose tissue.

Subcutaneous WAT biopsy specimens were obtained previously and 15 RNA samples were provided for retrospective analysis. All participants completed a screening evaluation that included a medical history and physical examination, standard blood tests, hemoglobin A_1c_, an oral glucose tolerance test, and assessment of intrahepatic TG content by MRI (31). A second set of RNA (*n* = 12) isolated from subcutaneous WAT biopsy specimens (12) was also used for analysis of gene expression. The study included obese individuals (BMI 38.3 ± 1.5; fasting plasma glucose 82 ± 3 mg/dL; HOMA-IR 2.2 ± 0.3) and people with recently diagnosed T2DM (BMI 35.2 ± 3.8; fasting plasma glucose 126 ± 31 mg/dL; HOMA-IR 7.8 ± 2.1). All participants provided written informed consent.

### Mass spectrometry.

Complete details of proteomic profiling have been described previously (63). Briefly, adipose tissues were homogenized in lysis buffer. After denaturation and trypsinization, peptides were extracted by 50% acetonitrile/0.1% formic acid and 80% acetonitrile/0.1% formic acid solution. The WAT proteome was profiled by nano-liquid chromatography–tandem mass spectrometry analysis with a nano-LC1000 coupled to a Thermo Q-Exactive (Thermo Fisher Scientific). Obtained MS-MS spectra were searched against the target-decoy mouse Reference Sequence database in the Proteome Discoverer 1.4 interface (PD1.4; Thermo Fisher Scientific) with the Mascot algorithm (Mascot 2.4; Matrix Science). The relative amount was calculated by the intensity-based absolute quantification algorithm and normalized to the intensity-based fraction of the total.

### qPCR.

Total RNA was extracted using the Direct-zol RNA MiniPrep kit (Zymo Research, R2051). cDNA was synthesized using qScript (QuantBio, 95048-100). Relative mRNA expression was measured with SsoAdvanced Universal Probes Supermix reactions (Bio-Rad, 175284) read out with a QuantStudio 3 real-time PCR system (Applied Biosystems). TATA-box binding protein (*Tbp)* was the invariant control. Roche Universal Probe Gene Expression Assays were used as previously described (38).

The TaqMan Advanced miRNA cDNA Synthesis Kit (Thermo Fisher Scientific, A28007) was used to synthesize miRNA cDNA from 20 ng total RNA. To extend mature miRNAs, polyadenylation and adaptor sequence ligation of the 3′ and 5′ ends, respectively, occur before universal priming and reverse transcription. To address low-expressing targets, cDNA is amplified by primers that recognize sequences appended to both ends, effectively minimizing amplification bias. Next, the TaqMan Advanced miRNA Assays (Thermo Fisher Scientific, A25576) were used to quantify relative gene expression. As recommended by the manufacturer, invariant RNA controls included *miR-423-3p*, *miR-451*, and *miR-423-5p*. The qSTAR microRNA Detection Assays (Origene) were also used to determine and verify absolute miRNA levels.

### Immunoblotting.

Cell and tissue lysates were prepared in Protein Extraction Reagent (Thermo Fisher Scientific) supplemented with Halt Protease and Phosphatase Inhibitor Cocktail (Thermo Fisher Scientific). Western blotting was performed with whole-cell lysates run on 4%–12% Bis-Tris NuPage gels (Life Technologies, Thermo Fisher Scientific) and transferred onto Immobilon-P Transfer Membranes (MilliporeSigma), followed by antibody incubation. Immunoreactive bands were visualized by chemiluminescence. Antibodies used in this study include HSP90 (Cell Signaling Technology, 4877), PPARg (Cell Signaling Technology, 2443), ADIPOQ (GeneTex, GTX112777), UCP1 (Abcam, ab10983), CYCS (Cell Signaling Technology, 4280), Cre (Cell Signaling Technology, 15036), FABP4 (Genetex, 116036), PAI-1 (Novus, NBP19223), COL1A1 (Genetex, 112731), and β-actin (MilliporeSigma, A5441). For tissue cytokine analysis, tissue lysates from 4 samples were pooled (125 μg/sample) and incubated with membranes from the Proteome Profiler Mouse Adipokine Array Kit (R&D Systems, ARY013).

### Glucose and insulin tolerance tests.

Mice were fed a 60% HFD (Bio-Serv) for 12–18 weeks before phenotyping. To determine glucose tolerance, mice were fasted 16 hours, and glucose was administered at 1.5 g/kg body weight by i.p. injection. To determine insulin tolerance, mice were fasted 4 hours before i.p. insulin injection (1.5 U/kg body weight). Blood glucose levels were measured by handheld glucometer.

### Analysis of hormone and lipid profiles.

Plasma insulin levels were quantified using ELISA (MilliporeSigma, EZRMI-13K). Free fatty acids (Zen-Bio GFA-1), leptin (Crystal Chem, 90030), and adiponectin (Thermo Fisher Scientific, KMP0041) were measured using commercially available kits.

### Hepatic TGs and cholesterol.

Tissue samples were analyzed for TGs (Triglyceride reagent; TR22421, Thermo Fisher Scientific) and cholesterol (Total Cholesterol Reagent; TR13421, Thermo Fisher Scientific) using liver homogenates mixed with a 1:2 chloroform/methanol solution followed by isolation of the lipid-rich chloroform layer (modified Folch method).

### Indirect calorimetry.

Mice were maintained on experimental diets and housed at room temperature in CLAMS-HC (Columbus Instruments). Oxygen consumption, CO_2_ emission, energy expenditure, food and water intake, and activity were measured for 5 days (BCM Mouse Metabolic and Phenotyping Core). Mouse body composition was examined by EchoMRI (Echo Medical Systems) before indirect calorimetry.

### In vitro experiments.

Subcutaneous human preadipocytes (Zen-Bio) were differentiated and transfected as previously described (19). SVF cells were isolated from mouse iWAT. Fat depots were digested in PBS containing collagenase I (1.5 U/mL; Roche, 17100-017) and dispase II (2.4 U/mL; MilliporeSigma, D4693) supplemented with 10 mM CaCl_2_ at 37°C for 40–45 minutes. The primary cells were filtered twice through 70 μm cell strainers and centrifuged at 700 rcf to collect the SVF. The SVF cell pellets were rinsed and plated. Adipocyte differentiation was induced by treating confluent cells in DMEM/F12 medium containing GlutaMAX (Thermo Fisher Scientific, 10565-018), 10% FBS, with 0.250 mM isobutylmethylxanthine (MilliporeSigma, 13347), 1 mM rosiglitazone (Cayman Chemical Co., 71740), 1 mM dexamethasone (Tocris Biosciences, 1126), 850 nM insulin (MilliporeSigma, I5500), and 1 nM T3 (MilliporeSigma, T-074). Four days after induction, cells were switched to the maintenance medium containing 10% FBS, 1 mM rosiglitazone, 1 mM dexamethasone, 850 nM insulin, and 1 nM T3. Experiments occurred 8–10 days after induction of differentiation, and ORO (Biovision, K580) was used to assess overall lipid accumulation. Adv-GFP and Adv-iCre infections were performed in *miR-30a^L/L^* inguinal WAT SVF cells to confirm transgenic expression of *miR-30a* in vitro. Adv-GFP and Adv-iCre were provided by the Gene Vector Core at Baylor College of Medicine.

### Microscopy and histology.

Mitochondria were labeled using MitoTracker CMX-ROS (Thermo Fisher Scientific, M7512). Live cells were pulsed with 500 nM MitoTracker for 15 minutes. Mitochondrial labeling was followed by cell fixation in 4% paraformaldehyde. Ammonium chloride was used to quench auto-fluorescence derived from residual paraformaldehyde. Fixed cells were incubated with guinea pig anti–perilipin 1 (Progen, GP-29) primary antibody at room temperature for 2 hours, then washed in 1× PBS containing 0.2% Triton X-100 (PBS-T) 3 times (5 min/wash), followed by incubation with Alexa Fluor 647–conjugated donkey anti–guinea pig IgG (Jackson ImmunoResearch Labs, 706=605-148). DAPI (MilliporeSigma, D8417), and LipidTOX (Life Technologies, Thermo Fisher Scientific, H34475) were used for nuclei and lipid-droplet labeling, respectively. Imaging was performed with the DeltaVision Core Image Restoration Microscope (GE Healthcare).

High content analysis was performed on human adipocytes cells plated and differentiated in 96-well plates (PerkinElmer; PhenoPlate). After differentiation, cells were stained live with BODIPY for 20 minutes at 37°C. The stains were removed, and cells were fixed with 4% paraformaldehyde at room temperature for 20 minutes. After fixation, cells were washed 3 times in PBS at room temperature for 3 minutes each. Finally, cells were stained with DAPI in a PBS solution containing 1% BSA and 0.1% TX-100 for 20 minutes. Cells were then washed 3 times with PBS at room temperature for 3 minutes each. Cells were left in PBS to be imaged on a Yokogawa CV8000 spinning disk high-throughput confocal microscope with maximum projection intensity images collected for each channel. We collected 15 fields per well. Images were analyzed using CellProfiler (37) and intensity measurements extracted to describe lipid features for each well.

Formalin-fixed, paraffin-embedded adipose tissue sections were stained for Sirius Red by the BCM Human Tissue Acquisition and Pathology Core. For immunohistochemical analysis of WAT, sections were stained using anti-Mac3 (BD Pharmingen, 550292). Sections of liver tissue were frozen in Tissue-Tek OCT compound (4583, Sakura Finetek USA), and neutral lipids were stained with ORO. Four ×20 fields of view per tissue were imaged using a Nikon Ci-L Bright-field microscope. Fiji software quantified adipocyte morphometry in histological sections of WAT.

Immunofluorescence staining of WAT was performed on sections from formalin-fixed and paraffin-embedded tissues. After deparaffinization in xylenes and ethanol, EDTA-based antigen retrieval was performed in a steamer for 30 minutes, followed by washing with 0.3% Triton X-100 and protein blocking in Protein Block (Dako). Primary antibodies (4°C for 12 hours) and secondary antibodies (room temperature for 1 hour) were applied to slides upon dilution with PBS containing 0.05% Tween 20. The antibodies were rabbit anti-CD11c (BD Pharmingen, 553802); mouse anti-CD206 (Abcam, ab8918,); Alexa Fluor 488 AffiniPure F(ab′)_2_ Fragment Donkey Anti–Rabbit IgG (Jackson ImmunoResearch Laboratories, 711546152), and Alexa Fluor 594 AffiniPure Donkey Anti–Mouse IgG (Jackson ImmunoResearch Labs, 715585150). Nuclei were visualized with DAPI staining. Immunofluorescence images were captured with a Zeiss LSM 710 confocal microscope and analyzed using ImageJ software.

### Luciferase reporter assays.

We used transient transfection methods to express *PAI-1* 3′-UTR luciferase fusions (Active Motif) in differentiated human adipocytes (Zen-Bio). miRNA binding to control regions (*RO1* 3′ UTR) of the *PAI-1* 3′-UTR was determined using LightSwitch assay reagents (Active Motif).

### FACS analysis of the WAT SVF.

Minced adipose tissues were placed in digestion buffer containing 0.5% BSA and 1 mg/mL collagenase (MilliporeSigma, C2139) and incubated in a 37°C shaking water bath for 30 minutes. The mixture was passed through a 100 μm filter before low-speed centrifugation. Erythrocytes were removed from the SVF pellet with RBC Lysis Buffer (BioLegend, 420301). The purified SVF pellet was resuspended in FACS buffer incubated with Fc Block (eBioscience, 14-0161-85) and stained with conjugated antibodies. The following antibodies were used for FACS: CD45 (eBioscience, 12-0481-82), F4/80 (BioLegend, 123113), and CD3 (BioLegend, 100312). Stained cells were washed twice in PBS and fixed in 1% formaldehyde before analysis. Samples were profiled using an LSRII cytometer (Becton Dickinson) coupled with FACS Diva (BD Biosciences) and FlowJo (Tree Star) software.

### Seahorse assays.

Respiration was measured in cultured adipocytes using an XF24 analyzer (Agilent). SVF cells were plated into V7-PS plates and grown to confluence. Cells were differentiated for 8–10 days. For the assay, medium was replaced with assay medium: 37°C unbuffered DMEM containing 4.5 g/L glucose, sodium pyruvate (1 mmol/L), and l-glutamine (2 mmol/L). Measurements were made at 37°C using 2-minute mix, wait, and measure intervals. Basal respiration was defined before sequential addition of oligomycin, carbonyl cyanide-4-(trifluoromethoxy) phenylhydrazone, rotenone, and antimycin A.

### Reverse phase protein arrays.

Protein lysates were prepared by the BCM Antibody-Based Proteomics Core for reverse phase protein arrays. The Aushon 2470 Arrayer (Aushon BioSystems) with a 40-pin (185 μm) configuration was used to spot lysates onto nitrocellulose-coated slides (Grace Bio-Labs). The slides were probed with 220 antibodies against total and phospho-proteins using an automated slide stainer (Dako). Primary antibody binding was detected using a biotinylated secondary antibody followed by streptavidin-conjugated IRDye 680 fluorophore (LI-COR). Fluorescent-labeled slides were scanned on a GenePix AL4200, and the images were analyzed with GenePix Pro 7.0 (Molecular Devices). Background-subtracted total fluorescence intensities of each spot were normalized for variation in total protein (Sypro Ruby) and nonspecific labeling.

### Hydroxyproline measurements.

Metabolites were extracted from homogenized tissues using a liquid-liquid extraction procedure described earlier (64). A sample pool was used as quality control. The extracted samples were then injected into the liquid chromatography-mass spectrometry system for analysis. Hydroxyproline was measured using targeted metabolomics via single-reaction monitoring in positive electrospray ionization mode, separated on XBridge Amide HPLC column (3.5 μm, 4.6 × 100 mm; Waters). The mobile phases consisted of (a) water with 0.1% formic acid (FA) and (b) acetonitrile with 0.1% FA. The flow rate was maintained at 0.3 mL/min throughout the analysis. The acquired mass spectra were processed and analyzed using Agilent MassHunter Quantitative Analysis Software, where peaks corresponding to hydroxyproline were identified and integrated. The quantified peak areas were normalized using a spiked internal standard.

### Statistics.

All measurements were taken from distinct biological samples. Unless otherwise noted, all statistical analyses were performed using GraphPad Prism 9 (GraphPad Software). In the case of multiple groups, 1- or 2-way ANOVA with post hoc tests were used to determine statistical significance. When only 2 groups were compared, *t* tests were used to determine statistical significance. Comparisons and replicate numbers are listed in each figure legend. Statistical analysis of energy balance was performed by analysis of covariance (ANCOVA) with lean body mass as a covariate (65). No statistical method was used to predetermine sample size. All data are expressed as the mean ± SEM, unless otherwise specified, and experiments were repeated at least 1 time.

### Study approval.

All animal procedures were approved by the Baylor College of Medicine IACUC. Human studies were approved by the Human Research Protection Office of Washington University School of Medicine in St. Louis (ClinicalTrials.gov NCT02706262) and the Baylor College of Medicine IRB (H-28439). Written informed consent was obtained from all participants before enrollment in the studies.

### Data availability.

All data are available in the Supporting Data Values file and are available upon request.

## Author contributions

SMH conceived and supervised the study. PKS and SMH conducted most of the experiments and mouse phenotyping. ARC, RS, and HW performed studies of immune cells and inflammation in adipose tissues. SMH, JBF, CERB, and MJB carried out the in vitro experiments and analysis of gene expression in adipocytes. RH, XL, and KS carried out the immunofluorescence and analysis of adipose tissue sections. KHK performed analysis of liver lipid profiles. SYJ implemented and prepared the proteomics studies of adipose tissues. SK provided human samples and helped with data interpretations. KHK designed the graphical abstract. SMH wrote the manuscript. All authors provided editorial input and reviewed and approved the final version of the article.

## Supplementary Material

Unedited blot and gel images

Supporting data values

## Version 1 06/05/2025

In-Press Preview

## Version 2 08/01/2025

Electronic publication
