# Supplementary material for: The microRNA miR-30a blocks adipose tissue fibrosis accumulation in obesity
Source: J Clin Invest. 2025 Jun 5;135(15):e175566. doi: 10.1172/JCI175566 (PMC12321386; doi:10.1172/JCI175566)

Full unedited gel for Figure 1A (Southern blot)

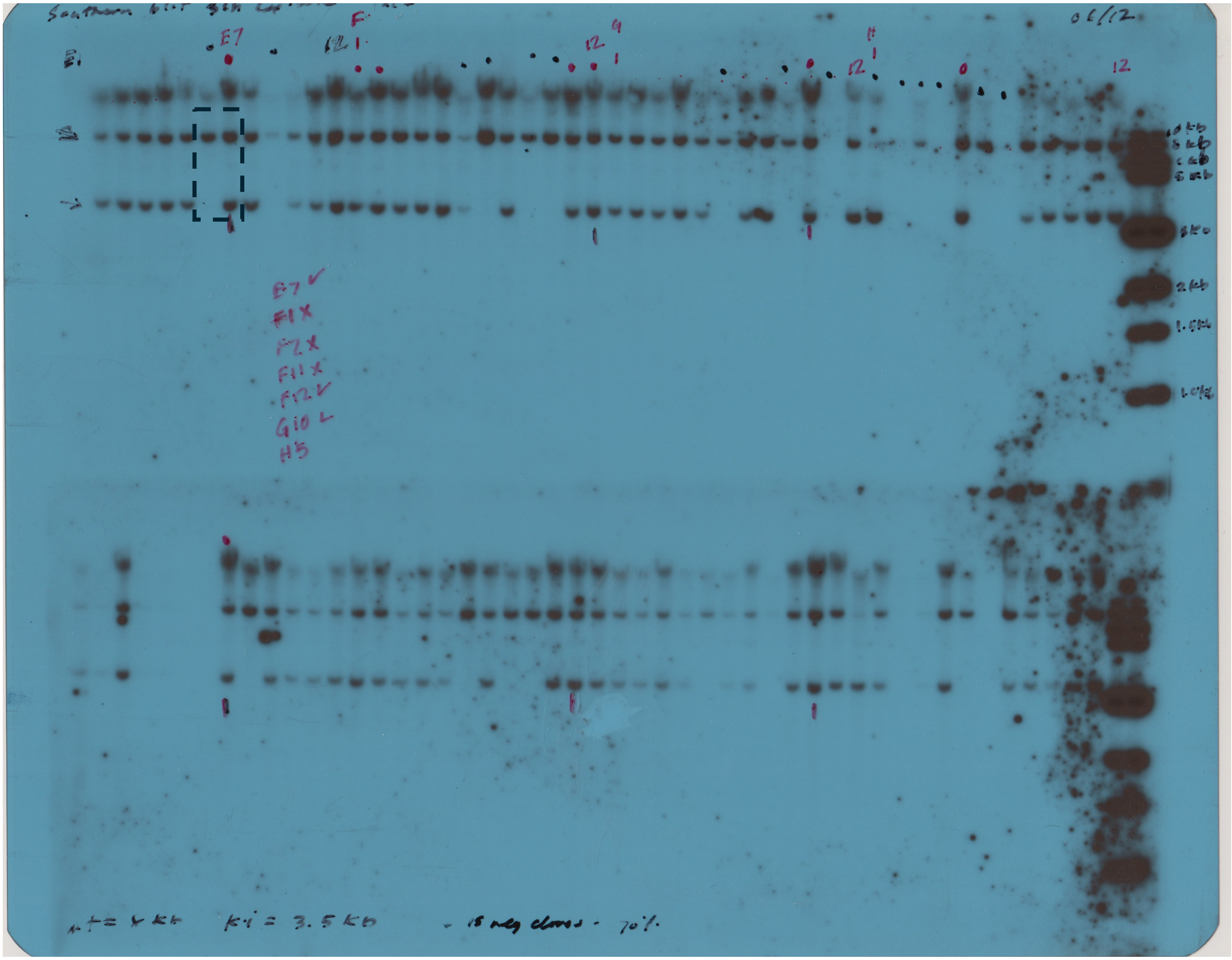

Full unedited gels for Figure 1D (Western blot)

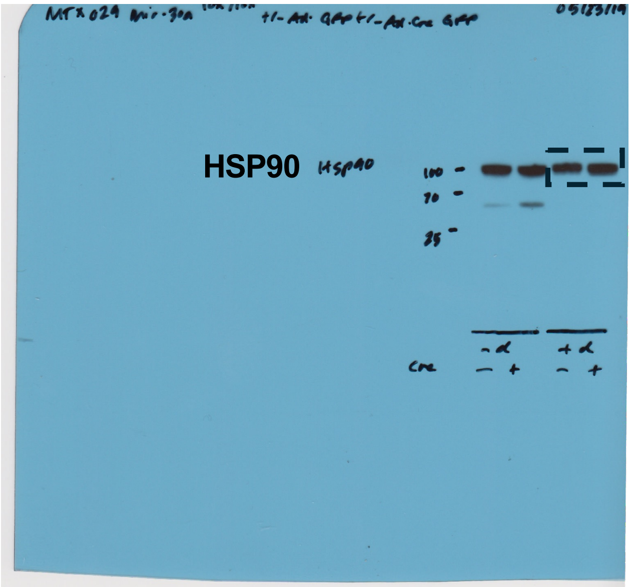

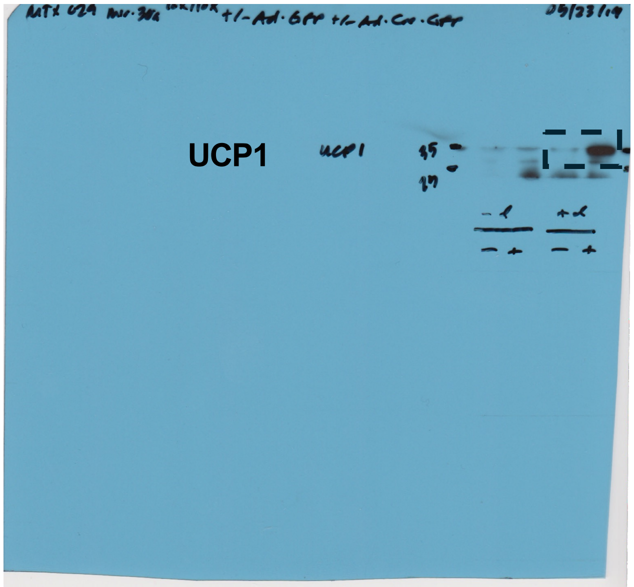

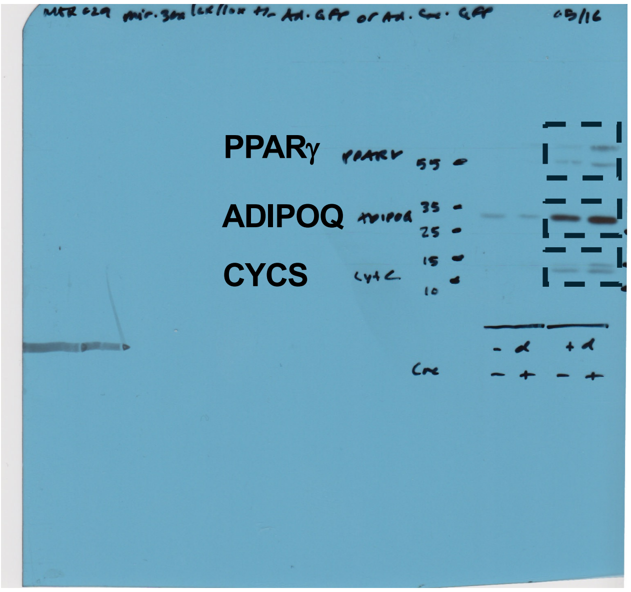

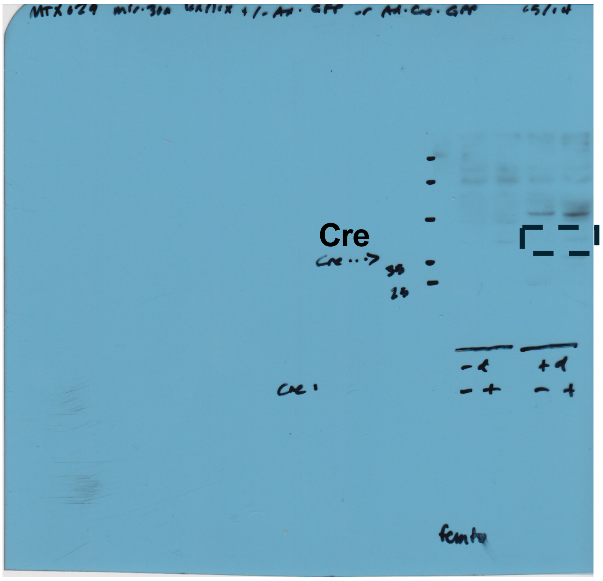

Full unedited gels for Figure 4B (adipokine arrays)

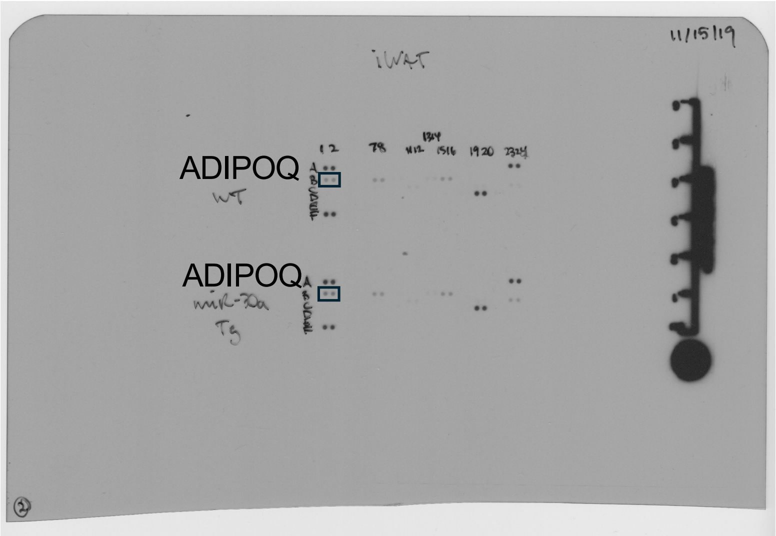

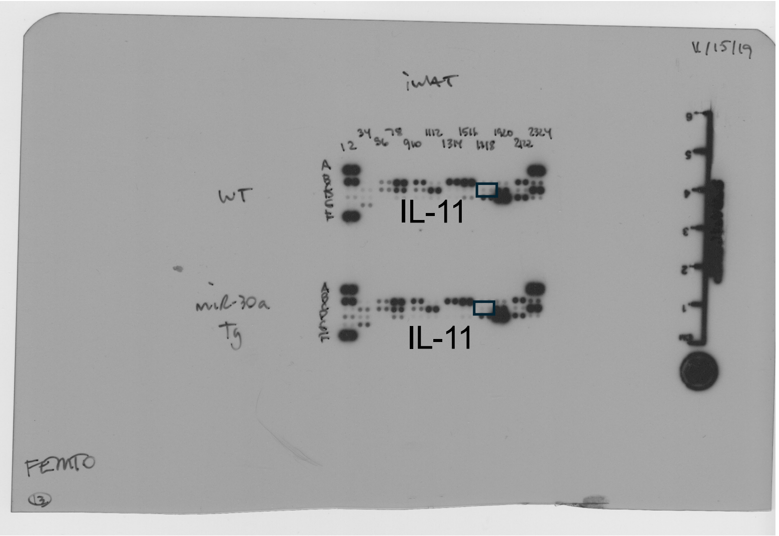

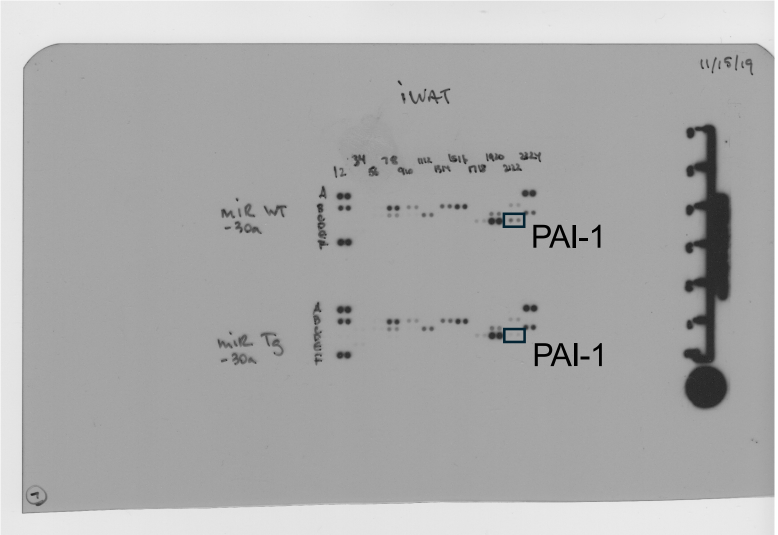

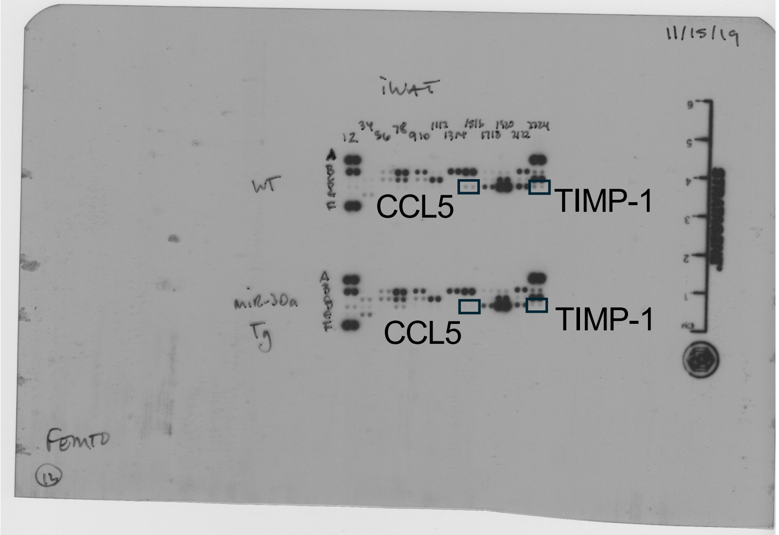

Full unedited gels for Figure 4I (Western blot)

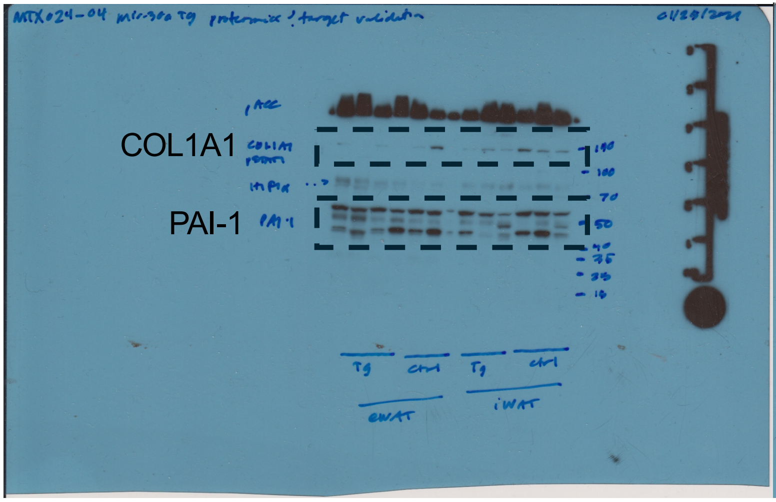

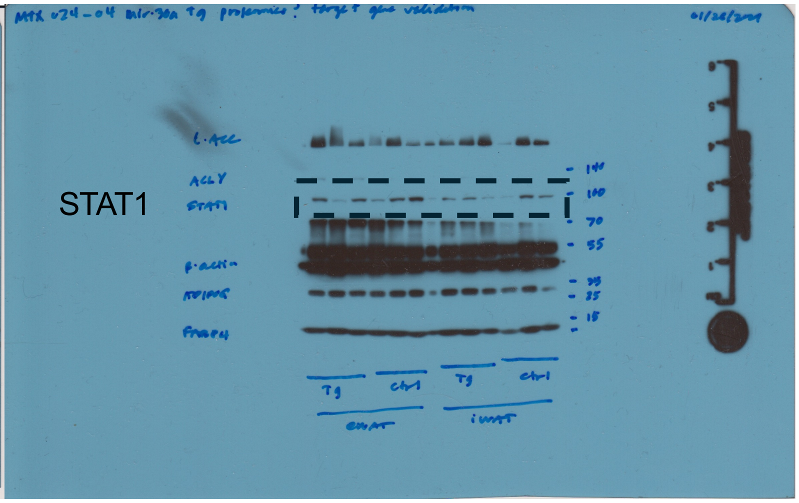

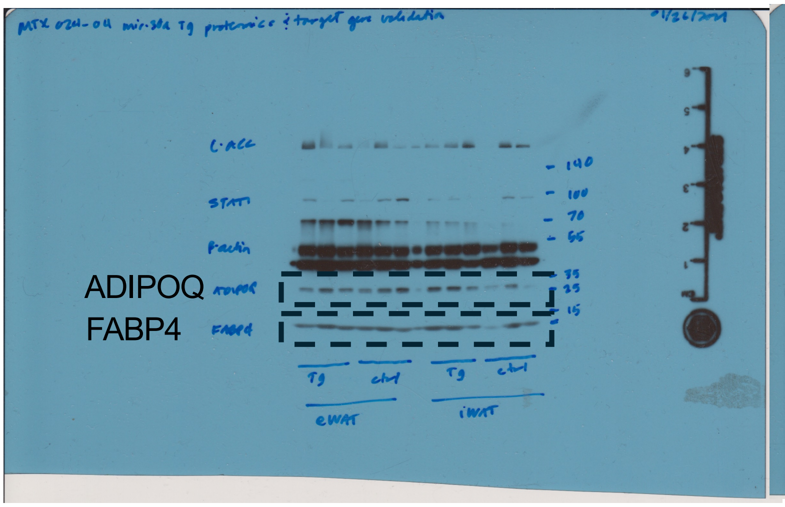

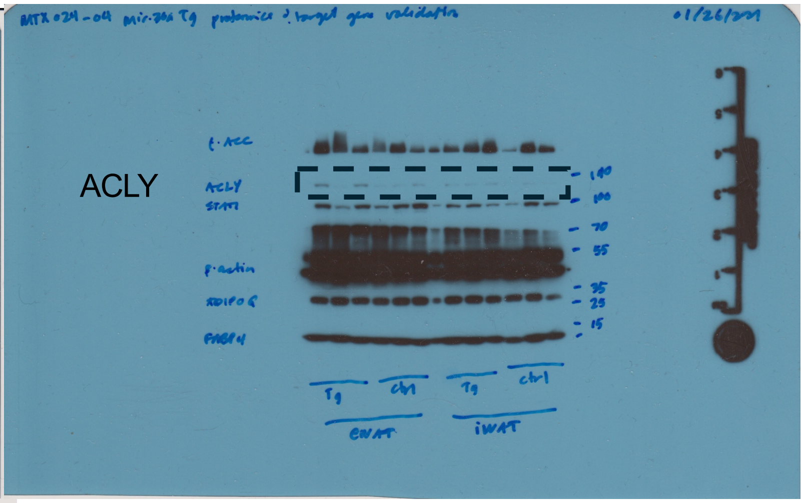

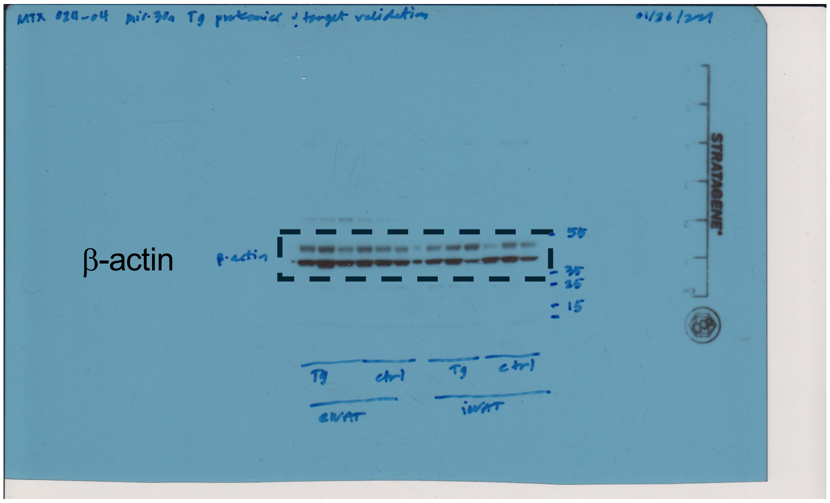

Full unedited gels for Figure 5C (Western blot)

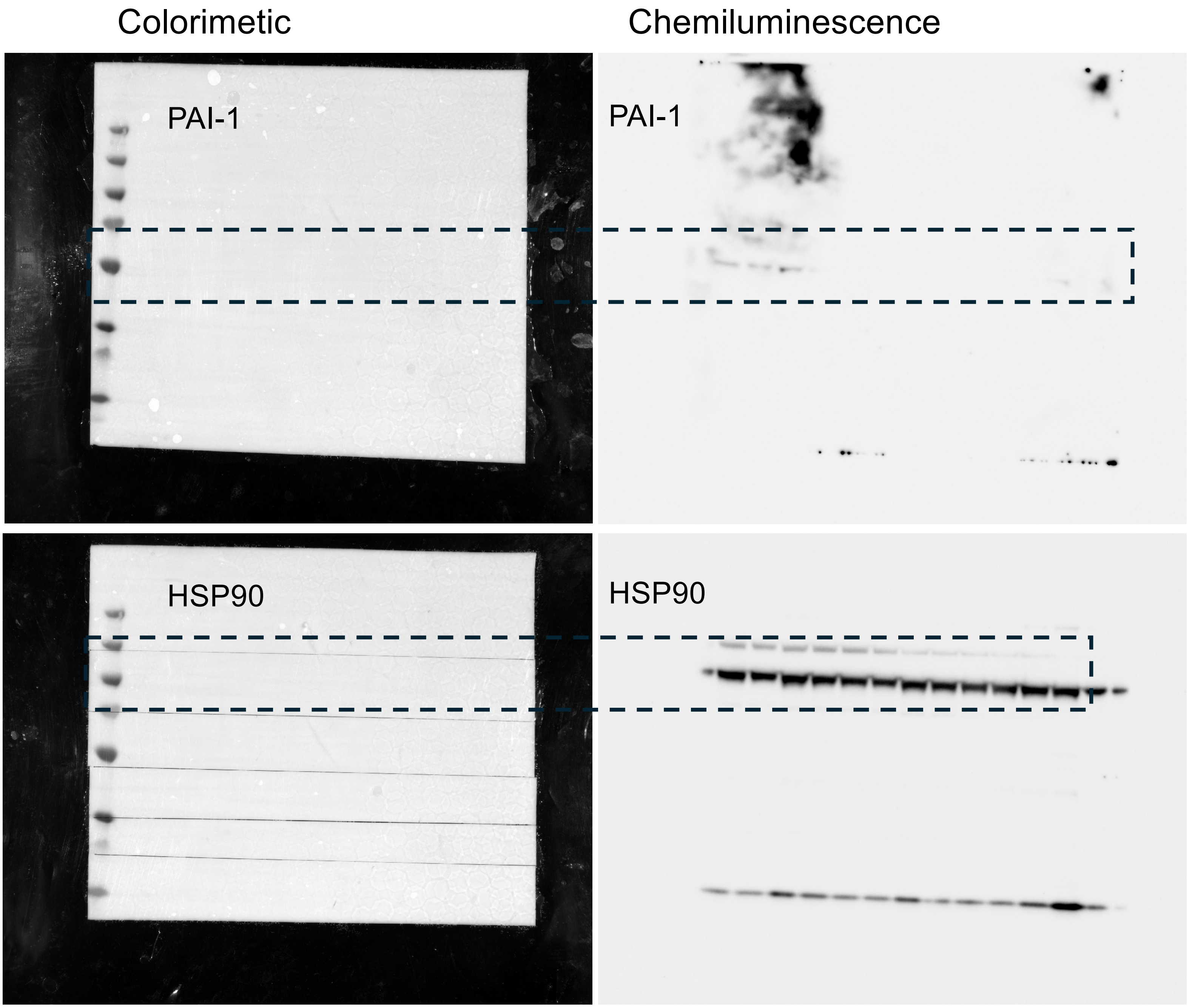

Supplement: Unedited blot and gel images [file jci-135-175566-s221.pdf]
